# Urate-lowering therapy, serum urate, inflammatory biomarkers, and renal function in patients with gout following pegloticase discontinuation

**DOI:** 10.1186/s13075-024-03318-5

**Published:** 2024-04-12

**Authors:** Emily E. Holladay, Amy S. Mudano, Fenglong Xie, Jingyi Zhang, Ted R. Mikuls, Brian LaMoreaux, Lissa Padnick-Silver, Jeffrey R. Curtis

**Affiliations:** 1 University of Alabama at Birmingham, 1825 University Blvd, Birmingham, AL 35233 USA; 2 Foundation for Advancing Science, Technology, Education, and Research, Birmingham, AL USA; 3 University of Nebraska Medical Center and the VA Nebraska-Western Iowa Health Care System, Omaha, NE USA; 4 Horizon Therapeutics Plc (Now Amgen Inc.), Deerfield, IL USA

**Keywords:** Gout, Pegloticase, Treatment gap, Restart, Discontinuation

## Abstract

**Background/Purpose:**

Little is known about long-term clinical outcomes or urate-lowering (ULT) therapy use following pegloticase discontinuation. We examined ULT use, serum urate (SU), inflammatory biomarkers, and renal function following pegloticase discontinuation.

**Methods:**

We conducted a retrospective analysis of gout patients who discontinued pegloticase using the Rheumatology Informatics System for Effectiveness (RISE) registry from 1/2016 to 6/2022. We defined discontinuation as a gap ≥ 12 weeks after last infusion. We examined outcomes beginning two weeks after last dose and identified ULT therapy following pegloticase discontinuation. We evaluated changes in lab values (SU, eGFR, CRP and ESR), comparing on- treatment (≤ 15 days of the second pegloticase dose) to post-treatment.

**Results:**

Of the 375 gout patients discontinuing pegloticase, median (IQR) laboratory changes following discontinuation were: SU: +2.4 mg/dL (0.0,6.3); eGFR: -1.9 mL/min (− 8.7,3.7); CRP: -0.8 mg/L (-12.8,0.0); and ESR: -4.0 mm/hr (-13.0,0.0). Therapy post-discontinuation included oral ULTs (86.0%), restarting pegloticase (4.5%), and no documentation of ULT (9.5%), excluding patients with multiple same-day prescriptions (*n* = 17). Oral ULTs following pegloticase were: 62.7% allopurinol, 34.1% febuxostat. The median (IQR) time to starting/restarting ULT was 92.0 days (55.0,173.0). Following ULT prescribing (≥ 30 days), only 51.0% of patients had SU < 6 mg/dL. Patients restarting pegloticase achieved a median SU of 0.9 mg/dL (IQR:0.2,9.7) and 58.3% had an SU < 6 mg/dL.

**Conclusion:**

Pegloticase treats uncontrolled gout in patients with failed response to xanthine oxidase inhibitors, but among patients who discontinue, optimal treatment is unclear. Based on this analysis, only half of those starting another ULT achieved target SU. Close follow-up is needed to optimize outcomes after pegloticase discontinuation.

## Rheumatology key messages

Information about gout treatment following pegloticase discontinuation is limited. We found that after discontinuing pegloticase, patients frequently used oral ULTs (86% of those discontinuing pegloticase). Approximately 77% started ULTs or restarted pegloticase within 6 months of pegloticase discontinuation. This gap in switching to another ULT suggests greater need for more optimal management of gout patients who discontinue pegloticase or have meaningful gaps in treatment.

Approximately 58% of patients who restarted pegloticase had a SU < 6 mg/dL as measured at a median (IQR) interval of 45.5 (39.8, 53.0) days. The context for pegloticase interruptions needs to be further explored.

## Introduction

Gout is an inflammatory arthritis initially treated with oral urate-lowering therapy (ULT), such as xanthine oxidase inhibitors (XOIs) or uricosuric agents [1–3]. However, some patients are unable to tolerate oral ULTs, have gout that is refractory to oral ULTs, or have contraindications to oral ULTs, leaving them vulnerable to gout sequelae and the associated disability and decreases in quality of life [2, 4, 5]. Pegloticase is a recombinant pegylated uricase approved by the US Food and Drug Administration (FDA) for treating refractory gout in patients who fail to respond to XOIs [6]. In most patients, pegloticase reduces serum urate levels (SU) < 6 mg/dL early in treatment which has been shown to reduce the occurrence of gouty attacks [4–8]. However, loss of treatment efficacy over time with pegloticase is relatively common, particularly in the absence of immunomodulatory co-therapy, and its discontinuation may be required if patients develop anti-drug antibodies (ADAs) which have been associated with loss of urate-lowering response and infusion reactions (IRs) [4, 5, 9–12].

The optimal gout treatment after pegloticase discontinuation has not yet been established, and little is known about long-term SU management following discontinuation. Additionally, more information is needed on how or if pegloticase influences renal function and systemic inflammation. Renal impairment is common in gout patients, and gout increases the risk of chronic kidney disease (CKD) [13–15]. This association is further complicated as CKD can limit the use of both oral ULTs and gout flare treatments. Further, there is a growing body of evidence that gout impacts systemic inflammation, highlighting the importance of examining inflammatory biomarker values in patients with gout [16, 17]. Here, we leveraged the American College of Rheumatology’s (ACR) Rheumatology Informatics System for Effectiveness (RISE) registry to explore post-pegloticase ULT treatment patterns and clinical laboratory outcomes.

## Methods

### Study design and data source

We conducted a retrospective analysis of patients with gout who received pegloticase using the ACR’s RISE electronic health record (EHR)-based registry. EHR data between January 2016 and June 2022 were examined. The ACR-RISE registry is the largest EHR data-derived rheumatology registry in the world [18]. It provides de-identified information on clinical measures including body mass index (BMI), SU, and other laboratory test results as well as diagnostic codes (e.g., ICD-10 codes), procedure codes, and medication information from over 2.4 million rheumatology patients of more than 1,000 rheumatology providers. Here, we examined pegloticase-treated patients with gout, focusing on various aspects of their health, including ULT use, SU, renal function (via estimated glomerular filtration rate [eGFR]), and inflammatory markers (C-reactive protein [CRP], erythrocyte sedimentation rate [ESR]) before, during, and after pegloticase therapy.

### Study population and exposure groups

Study inclusion required a diagnosis of gout, defined by the presence of at least one international classification of disease code (ICD) assigned by a rheumatology provider during an ambulatory visit (ICD-9: 274*, ICD-10: M10*, M1A*). The study population included patients ≥ 18 years of age at their first gout diagnosis recorded in the RISE data, with that diagnosis occurring prior to beginning pegloticase therapy. Healthcare Common Procedure Coding System (HCPCS) code J2507 was used to identify each pegloticase infusion within the RISE registry. Given the focus on laboratory results in this analysis, patients were required to have at least one on-treatment and one post-treatment value for SU, eGFR, CRP, or ESR, and patients who had no lab results available in the RISE data (e.g., because labs were measured via an outside laboratory) were excluded.

The end of pegloticase treatment was conservatively defined based on a gap of ≥ 12 weeks after a pegloticase infusion, informed by the typical infusion interval of every two weeks based on its labeled dosing. Following discontinuation, we examined outcomes beginning two weeks after the last pegloticase dose, defined as the index date.

### Outcome definitions

Baseline laboratory values, including SU, eGFR, CRP, and ESR, were defined as those measured prior to first pegloticase infusion. Estimated GFR was calculated using the race-free CKD-EPI Eq 19. Response to prior medication was defined as SU ≤ 6 mg/dL after the second prescription of a ULT. Given the rapidity of SU-lowering following pegloticase initiation [6], laboratory tests performed 1–15 days after the second pegloticase infusion were assumed to reflect on-treatment effects. As a comparison, laboratory tests beyond two weeks following the index date (i.e., pegloticase discontinuation) were defined as the post-pegloticase discontinuation values. We examined changes in laboratory value(s) (SU, eGFR, CRP, and ESR), comparing on-treatment and post-pegloticase discontinuation values. Additionally, the effect of ULT initiation following pegloticase discontinuation was examined using laboratory values obtained at least 30 days after starting any post-index oral ULT (allopurinol, febuxostat, lesinurad, or probenecid) or re-initiating pegloticase. The proportion of patients with SU < 6 mg/dL was examined based on the ACR-recommended treat-to-target SU value and treatment efficacy definitions in pegloticase clinical trials [5, 6, 11].

### Covariates

We measured summary statistics of patient characteristics. Response to alopurinol or febuxostat was analyzed using SU after two consecutive prescriptions of the same medication within 12 months and prior to the start of pegloticase or after pegloticase discontinuation. We measured age at index and calculated BMI using the most recent value prior to the index date. All available prior data were included in the history of covariate calculations for selected covariates including the frequency of gout diagnoses, RxRisk, and gout-related medication use at or before the first pegloticase infusion. RxRisk is a comorbidity index, based on medication history, to assess the prevalence of 46 comorbid conditions. The RxRisk score indicates the number of comorbidities a patient has. We identified gout-related medications (e.g., glucocorticoids, colchicine, and nonsteroidal anti-inflammatory drugs [NSAIDs]) used at or before the index date using various coding systems including the HCPCS, national drug code (NDC), prescription concept unique identifier (RXCUI) from the National Library of Medicine (NLM), generic product identifier (GPI), or generic/brand name.

### Analysis

We performed descriptive analyses to characterize the demographics and clinical history of the study sample. We calculated mean and standard deviation (SD) and median and interquartile range (IQR) for continuous variables and frequencies and percentages for categorical variables. We generated tables for the cohort and for ULT use after pegloticase discontinuation. Kruskal-Wallis rank sum tests examined differences in the distribution of characteristics between medication groups.

We analyzed post-treatment effects following pegloticase discontinuation using descriptive statistics for SU, eGFR, CRP, and ESR. Mean and SD and median and IQR were calculated for baseline, on-treatment, and post-pegloticase discontinuation values. We censored patients from post-ULT initiation analyses if they did not restart pegloticase or another ULT, or if patients were prescribed multiple ULTs on the same day.

Kaplan Meier curves assessed the probability of starting any ULT, including pegloticase re-initiation, after pegloticase discontinuation. We utilized a Sankey plot to analyze ULT medication changes after pegloticase discontinuation and censored patients who started multiple ULTs on the same day. Local regression locally estimated scatterplot smoothing (LOESS) curves analyzed the changes between post-pegloticase discontinuation and post-ULT initiation lab values.

All analyses were conducted using SAS 4 (SAS, Cary, NC). This study was reviewed and approved by the University of Alabama at Birmingham Institutional Review Board (Birmingham, AL). This study was classified as exempt, waiving the requirement of informed consent. All study conduct adhered to the tenets of the Declaration of Helsinki.

## Results

**Fig. 1:**
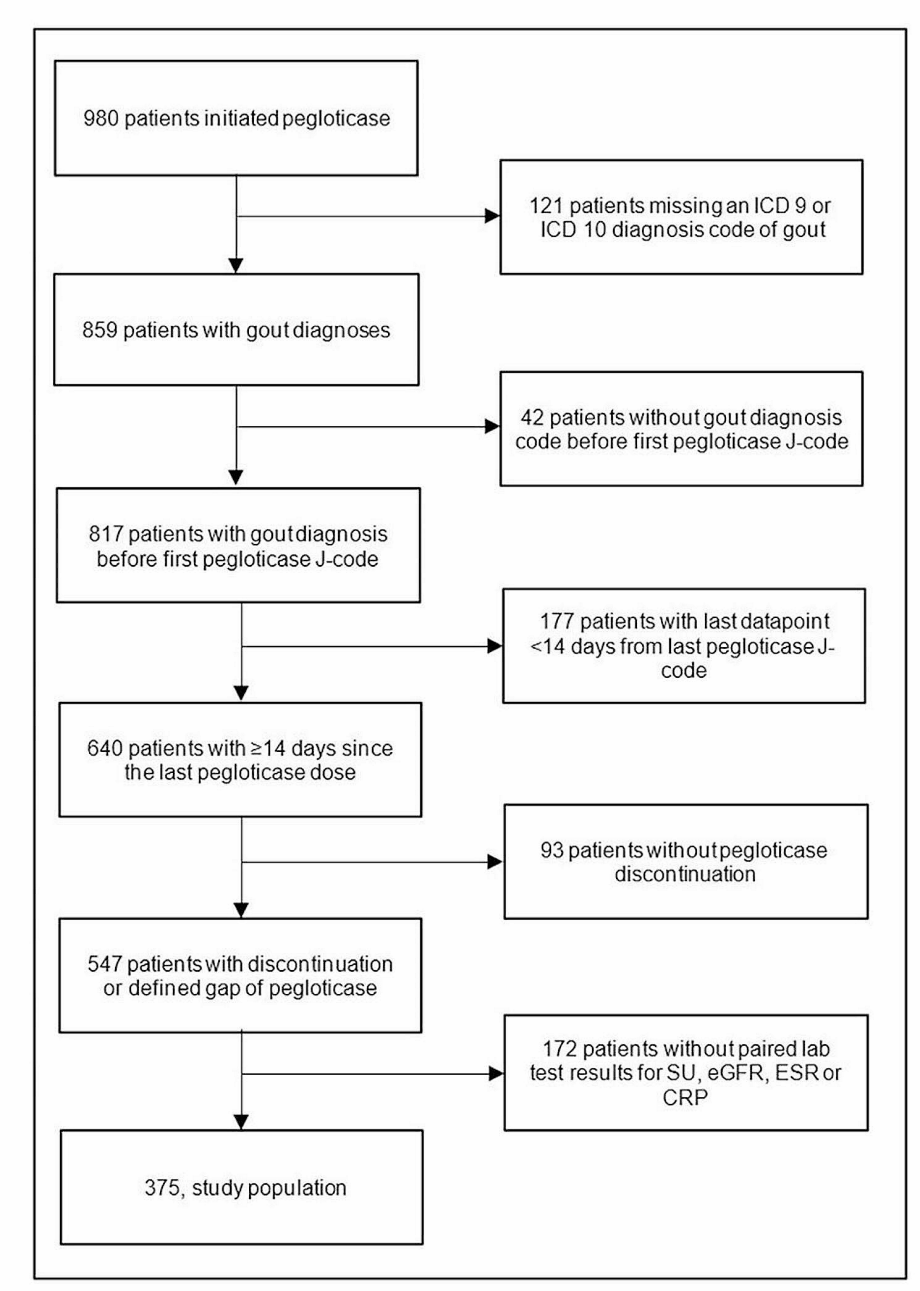
Attrition Table for the ACR-RISE Registry Pegloticase Discontinuation Cohort. ICD = international classification of disease code; SU = serum urate; eGFR = estimated glomerular filtration rate; ESR = erythrocyte sedimentation rate; CRP = C-reactive protein

After applying inclusion and exclusion criteria, we identified 375 patients with gout who discontinued pegloticase and had paired laboratory test results available (Fig. 1). The overall sample consisted mostly of male (82.9%), white (62.7%), non-Hispanic (64.8%) patients with tophi (76.8%) (Table 1). The mean (SD) age of patients was 60.3 ± 14.7 years, with a mean (SD) RxRisk of 9.0 ± 4.1 (scale: 0–46), and a mean BMI of 32.4 ± 8.7 kg/m^2^. In the 12 months prior to starting pegloticase, patients had prescriptions for up to 3 ULTs. Allopurinol was the most frequently prescribed ULT followed by febuxostat, probenecid, and lesinurad. Among the 55 previous allopurinol and 28 previous febuxostat users with 2 consecutive prescriptions for the same ULT and SU labs, response to these medications was infrequent (32.7% and 28.6%, respectively). The sample also included a large proportion of patients with use of NSAIDs (59.2%), glucocorticoids (83.2%), and colchicine (77.1%) on or before the index date. Conventional immunomodulator (IMM) use was also common (30.7%), with use attributed to pegloticase co-therapy (to reduce immunogenicity) or the treatment of concomitant comorbidities (e.g., psoriasis or an alternative rheumatic disease).

**Table 1:** Baseline Demographics of Gout Patients Discontinuing Pegloticase

		Overall (*N* = 375)
Age, Years, Mean (SD)		60.3 (14.7)
Male, n (%)		311 (82.9)
Race, n (%)		
White		235 (62.7)
Black or African American		34 (9.1)
Other or unknown		106 (28.3)
Ethnicity, n (%)		
Not Hispanic or Latino		243 (64.8)
Hispanic or Latino		10 (2.7)
Missing		122 (32.5)
BMI on or before the index date, kg/m^2^		
Mean (SD)		32.4 (8.7)
Missing, n		15
Number of gout diagnosis codes, Mean (SD)		14.4 (12.1)
RxRisk (0–46), Mean (SD)		9.0 (4.1)
Tophi, n (%)		288 (76.8)
**Medication use in the 15 months before pegloticase ** ^**a**^		
Allopurinol, n (%)		181 (71.0)
Febuxostat, n (%)		101 (39.6)
Probenecid, n (%)		16 (6.3)
Lesinurad, n (%)		9 (3.5)
None, n (%)		120 (32.0)
**Medication use on or before pegloticase discontinuation**		
Glucocorticoids, n (%)		312 (83.2)
Colchicine		289 (77.1)
NSAIDs, n (%)		222 (59.2)
Opioids, n (%)		122 (32.5)
IMMs, n (%)		115 (30.7)
Methotrexate, n (%)		93 (24.8)
Leflunomide, n (%)		13 (3.5)
Azathioprine, n (%)		16 (4.3)
Mycophenolate, n (%)		9 (2.4)
**Baseline laboratory values**		
SU, mg/dL ^b^		
Mean (SD)		7.8 (2.7)
Median (IQR)		8.4 (6.1, 9.8)
eGFR, mL/min/1.73m^2 c^		
Mean (SD)		74.8 (27.8)
Median (IQR)		74.5 (54.9, 97.0)
CRP, mg/L ^d^		
Mean (SD)		16.1 (41.1)
Median (IQR)		5.0 (2.0, 13.0)
ESR, mm/hr ^e^		
Mean (SD)		25.0 (23.8)
Median (IQR)		17.5 (8.0, 33.0)

SD = standard deviation; BMI = body mass index; IMMs = immunomodulatory medications; NSAID = non-steroidal anti-inflammatory drug; SU = serum urate; IQR= interquartile range; eGFR = estimated glomerular filtration rate; CRP = C-reactive protein; ESR = erythrocyte sedimentation rate

Note: RxRisk was calculated using all available data prior to the first pegloticase infusion. All other baseline characteristics were recorded using the most recent measurements prior to the first pegloticase infusion

^a^*N* = 251; Patients could have been prescribed multiple medications in the 12 months before starting pegloticase. Categories are not mutually exclusive

^b^*N* = 326

^c^*N* = 275

^d^*N* = 210

^e^*N* = 126

Opioid use at or before the index date was found in 122 (32.5%) patients. The median (IQR) baseline (pre- pegloticase treatment) SU, eGFR, CRP, and ESR laboratory values were 8.4 mg/dL (6.1, 9.8), 74.5 mL/min/1.73m^2^ (54.9, 97.0), 5.0 mg/L (2.0, 13.0), and 17.5 mm/hr (8.0, 33.0), respectively. In contrast, the on-treatment SU levels were appreciably lower. The median (IQR) SU level was 0.9 (0.2, 6.6) after a median (IQR) of 27.0 (25.0, 28.0) days into pegloticase treatment, consistent with pegloticase treatment response in most patients. However, the upper SU quartile of 6.6 mg/dL indicated that as many as 25% of patients had suboptimal SU-control, or were losing SU-lowering response, even early in the course of treatment.

The median (IQR) number of pegloticase infusions received was 8.0 (3.0, 14.0) with a maximum of 66 infusions. The median (IQR) duration of therapy (time between first and last infusion) was 112.0 days (33.0, 204.5) with a maximum of 1,240 days (Table 2). Approximately 73.1% of patients had an SU < 6 mg/dL during pegloticase treatment. The median SU increased to 5.8 mg/dL (IQR: 3.5, 8.3) after pegloticase discontinuation, a median (IQR) SU change of + 2.4 mg/dL (0.0, 6.3) compared to on-treatment SU values. Median (IQR) pegloticase on-treatment laboratory values were 74.5 mL/min/1.73m^2^ (54.0, 95.0), 8.0 mg/L (2.4, 24.7), and 23.5 mm/hr (6.8, 46.8) for eGFR, CRP, and ESR, respectively, after approximately 4 weeks of pegloticase treatment (median [IQR]:27.0 days [26.0, 28.0], 28.0 days [27.3, 29.0], and 28.0 days [27.8, 28.3]). Following pegloticase discontinuation, eGFR, CPR, and ESR changed by a median (IQR) of -1.9 mL/min/1.73m^2^ (-8.7, 3.7), -0.8 mg/L (-12.8, 0.0), and − 4.0 mm/hr (-13.0, 0.0), respectively, compared to on-treatment levels. Median post-pegloticase discontinuation values for SU, CRP, and ESR values were lower than pre-pegloticase laboratory values (Tables 1 and 2). However, a meaningful proportion of patients were missing clinical laboratory data (ranging from 4.5% for SU to 58.9% for ESR).

**Table 2:** Pegloticase Treatment Parameters and Observed Laboratory Values

Characteristic	Pegloticase treatment laboratory values ^a^ Overall (*N* = 375)	Post-pegloticase discontinuation laboratory values ^b^	Post-ULT initiation laboratory values ^c^
SU, mg/dL	(*N* = 253)	(*N* = 350)	(*N* = 257)
Mean (SD)	3.0 (3.9)	5.8 (3.5)	6.1 (2.5)
Median (IQR)	0.9 (0.2, 6.6)	5.8 (3.5, 8.3)	5.8 (4.6, 7.4)
eGFR, mL/min/1.73m^2^	(*N* = 98)	(*N* = 317)	(*N* = 233)
Mean (SD)	74.2 (26.8)	75.8 (27.5)	77.2 (26.8)
Median (IQR)	74.5 (54.0, 95.0)	77.5 (56.4, 96.2)	79.0 (56.5, 98.6)
CRP, mg/L	(*N* = 38)	(*N* = 110)	(*N* = 72)
Mean (SD)	18.4 (27.2)	10.3 (18.1)	7.4 (12.7)
Median (IQR)	8.0 (2.4, 24.7)	3.7 (1.4, 9.8)	3.3 (1.8, 8.2)
ESR, mm/hr	(*N* = 28)	(*N* = 67)	(*N* = 48)
Mean (SD)	28.4 (24.0)	21.5 (20.2)	21.7 (18.8)
Median (IQR)	23.5 (6.8, 46.8)	17.0 (6.0, 27.5)	18.0 (6.0, 32.3)
**Pegloticase treatment**			
Pegloticase infusions			
Median (IQR)	8.0 (3.0, 14.0)	-	-
≥ 12 pegloticase infusions, n (%)	147 (39.2)	-	-
Median (IQR) treatment length, days	112.0 (33.0, 204.5)	-	-
SU < 6 on-treatment, n (%)	185 (73.1)	-	-
N	253	-	-
Tophi, n (%)	-	257 (68.5)	-
**Laboratory value changes**			
		**On-treatment to post-pegloticase discontinuation**	**Post-pegloticase discontinuation to post-ULT initiation**
SU, mg/dL	-	(*N* = 252)	(*N* = 257)
Mean (SD)	-	2.8 (4.6)	0.1 (3.1)
Median (IQR)	-	2.4 (0.0, 6.3)	0.0 (-1.0, 0.5)
eGFR, mL/min/1.73m^2^	-	(*N* = 93)	(*N* = 233)
Mean (SD)	-	-2.8 (11.6)	-0.6 (10.2)
Median (IQR)	-	-1.9 (-8.7, 3.7)	0.0 (-3.7, 0.6)
CRP, mg/L	-	(*N* = 18)	(*N* = 72)
Mean (SD)	-	-4.2 (16.6)	-1.2 (15.5)
Median (IQR)	-	-0.8 (-12.8, 0.0)	0.0 (0.0, 0.0)
ESR, mm/hr	-	(*N* = 9)	(*N* = 48)
Mean (SD)	-	-9.0 (11.9)	-1.2 (11.9)
Median (IQR)	-	-4.0 (-13.0, 0.0)	0.0 (0.0, 0.0)
**Target SU**			
SU < 6 post-pegloticase discontinuation, n (%)	-	181 (51.7)	-
N	-	350	-
**ULT restart**			
SU < 6 after post-ULT initiation, n (%)	-	-	131 (51.0)
N	-	-	257
Time between stopping and restarting ULT, days			
Median (IQR)	-	-	92.0 (55.0, 173.0)
N	-	-	257

SD = standard deviation; IQR = interquartile range; SU = serum urate; CRP = C-reactive protein; ESR = erythrocyte sedimentation rate; eGFR = estimated glomerular filtration rate; ULT = urate-lowering therapy

Note: Oral ULTs should not be administered during pegloticase treatment (including the 2-week period following the last infusion)

^a^ On-treatment lab values were limited to 1–15 days after the second pegloticase infusion

^b^ Post-pegloticase discontinuation lab values were measured 2 weeks following the index date

^c^ Post-ULT lab values were measured > = 30 days after starting a ULT drug

**Fig. 2:**
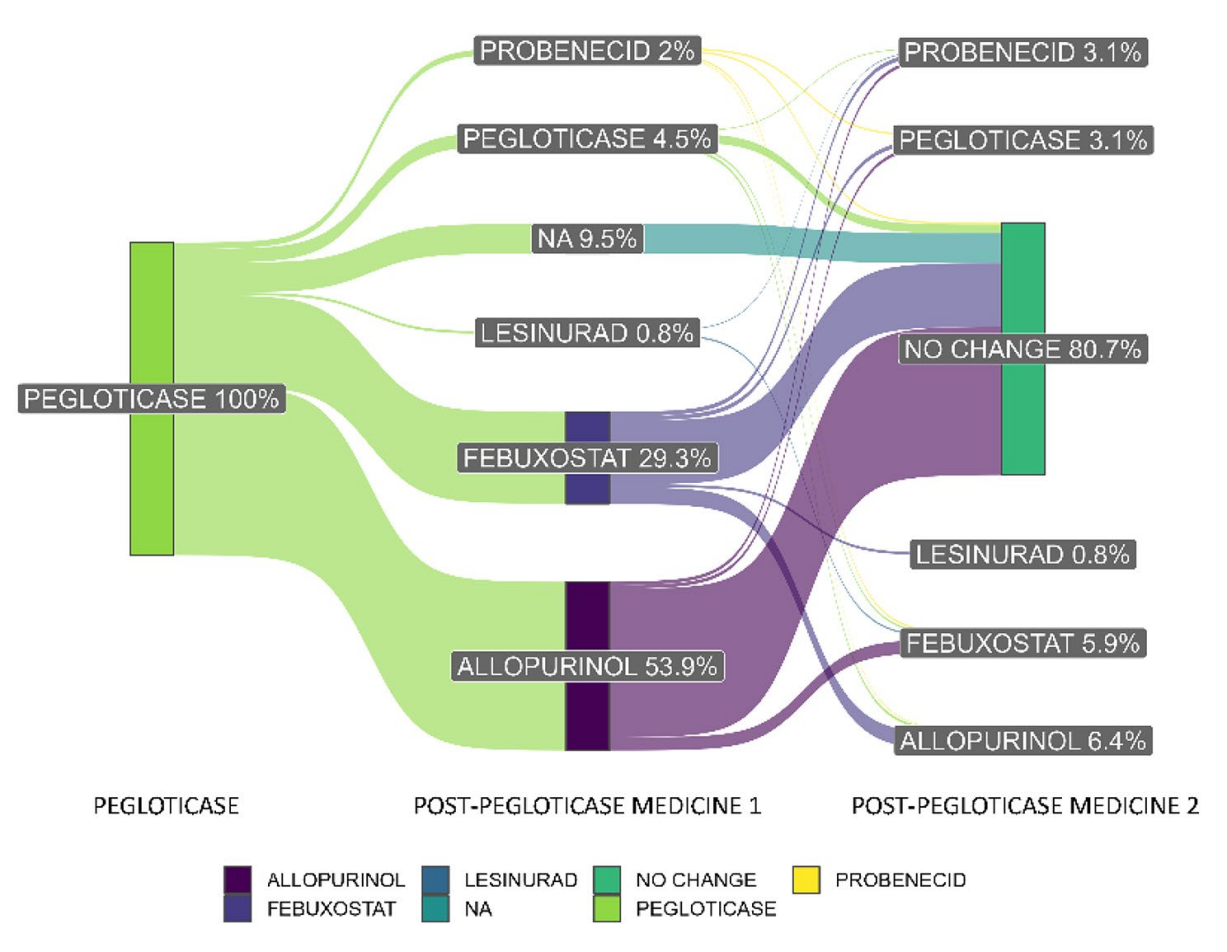
ULT Initiated After Pegloticase Discontinuation (*n* = 358^a^). ULT = urate-lowering therapy. ^a^ Patients prescribed multiple ULT’s for medicine 1 or 2, after pegloticase discontinuation, were censored

**Fig. 3:**
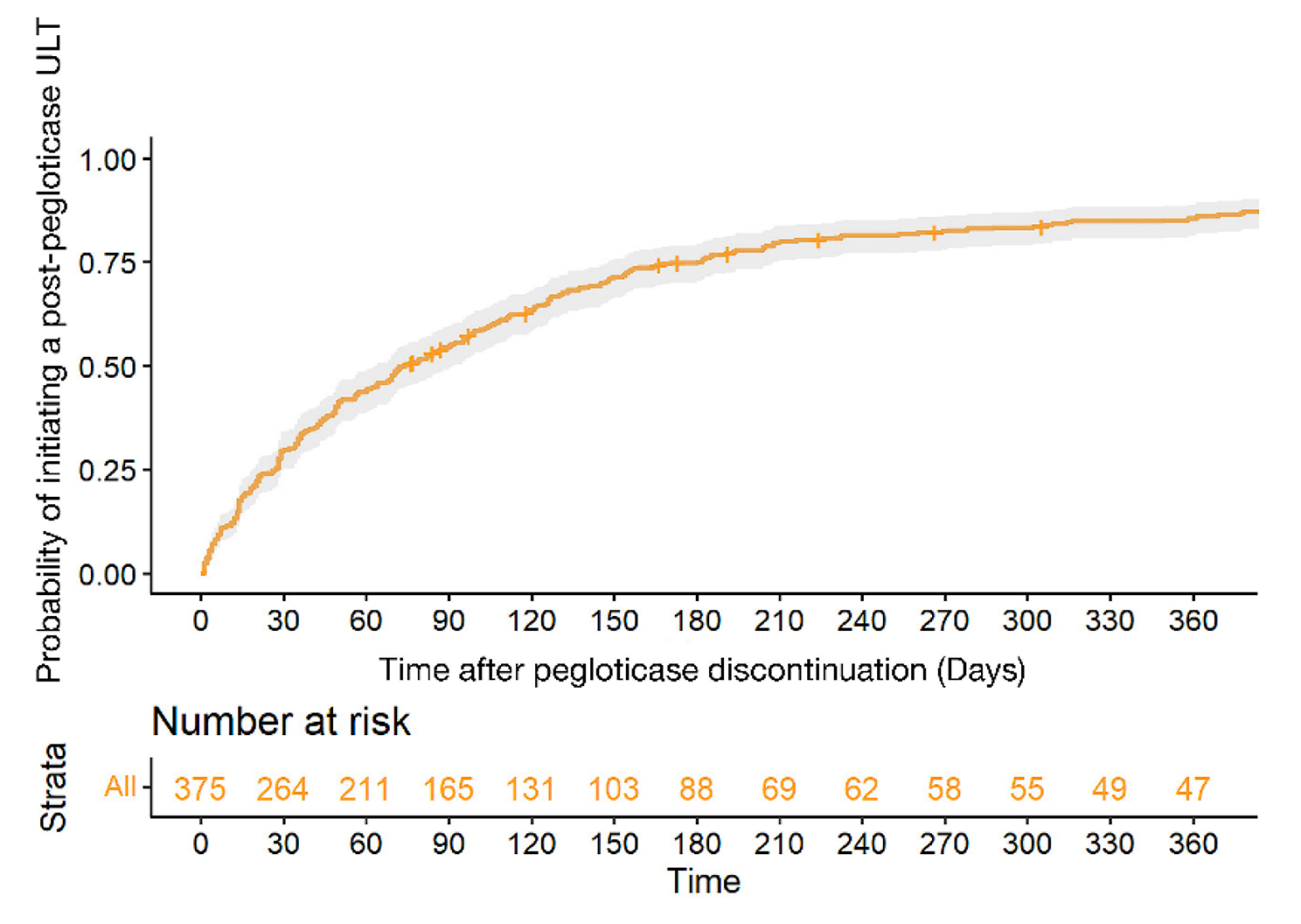
Probability of Starting a ULT Following Pegloticase Discontinuation. Pegloticase discontinuation is defined as 14 days following the last pegloticase infusion. The gray shaded region represents the 95% confidence interval. ULT = urate-lowering therapy

Following pegloticase discontinuation, subsequent gout management included beginning an oral ULT (86.0% of patients), restarting pegloticase (4.5%), or not receiving any ULT (9.5%) in the reported time period. Of those who initially started oral ULTs (*n* = 317), 62.7% used allopurinol, 34.1% used febuxostat, 2.2% used probenecid, and 1.0% used lesinurad. Some patients switched ULTs following pegloticase discontinuation, with their second ULT medication change also shown in Fig. 2. Kaplan Meier analysis showed 76.5% of patients had started oral ULTs or restarted pegloticase within 6 months after discontinuing pegloticase (Fig. 3).

Post-ULT laboratory values were measured at least 30 days after the first oral ULT start or the restart of pegloticase. There was a median (IQR) interval of 92 days (55.0, 173.0) between discontinuing pegloticase and restarting any ULT (Table 2). Following at least 30 days after transitioning to a non-pegloticase ULT, the proportion of patients with a SU < 6 mg/dL was 51.0%. Of those who restarted allopurinol or febuxostat following pegloticase discontinuation, 64.9% and 39.3%, respectively, had urate-lowering response (SU < 6 mg/dL). The median (IQR) time of SU measurement following the second allopurinol and febuxostat prescription was 57.0 days (1.0, 190.0) and 64.5 days (1.0, 273.5), respectively. The proportion of patients who restarted pegloticase and had a SU < 6 mg/dL was 58.3% as measured at a median (IQR) of 45.5 days (39.8, 53.0) after initially stopping pegloticase. Patients who restarted pegloticase (*n* = 16) achieved a median on-therapy SU of 0.9 mg/dL (IQR: 0.2, 9.7; all measures at least 30 days after restarting pegloticase), with 60.0% of patients receiving immunomodulator co-therapy.

The eGFR, CRP, and ESR values did not meaningfully or significantly change between post-pegloticase discontinuation levels and post-pegloticase ULT treatment levels. Further, changes in post-pegloticase discontinuation and post-pegloticase ULT initiation laboratory measures were not significantly different between patients treated with oral ULTs or pegloticase (not shown).

**Fig. 4:**
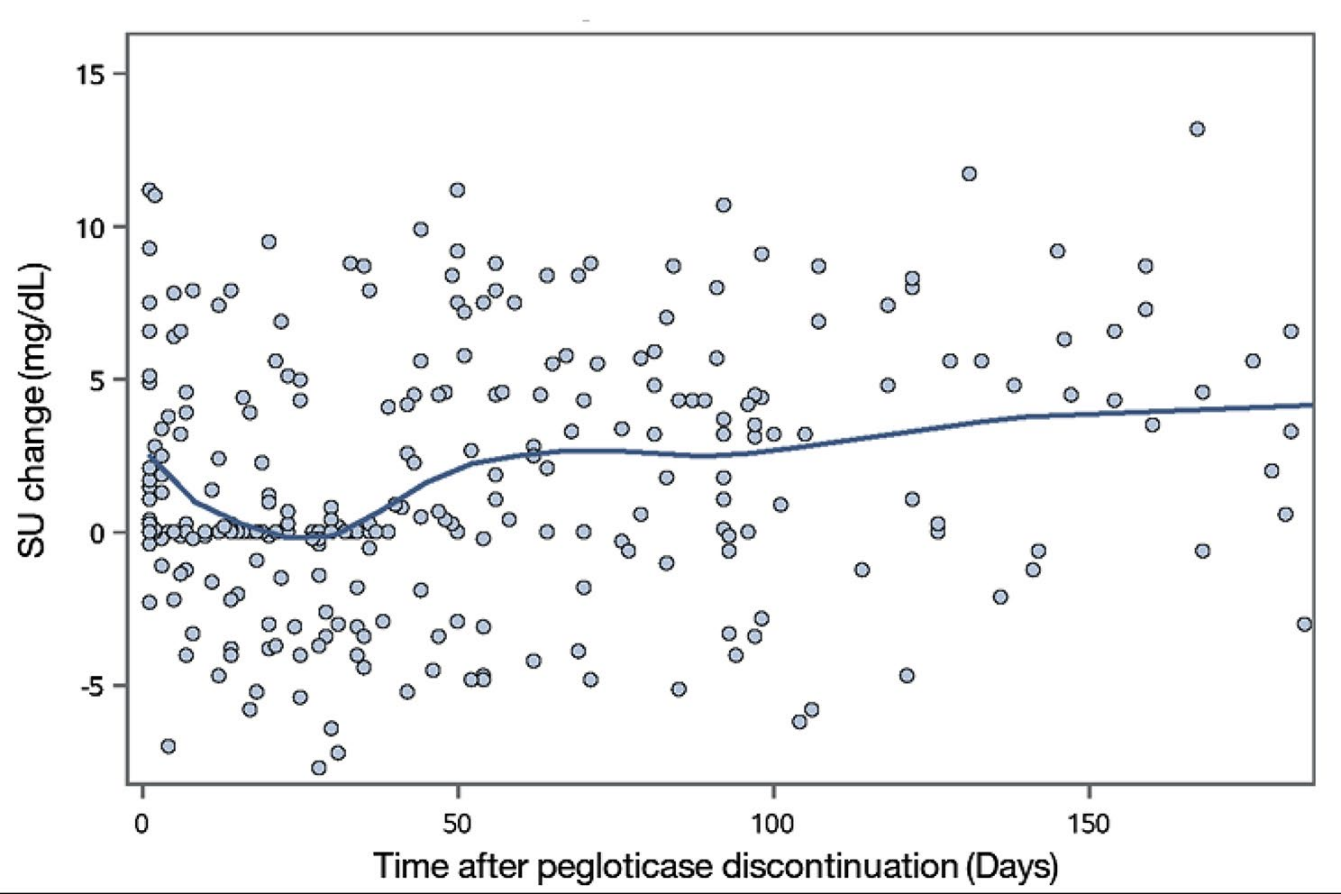
Changes in Laboratory Values Following Pegloticase Discontinuation. SU = serum urate

There was a high degree of variability in changes in SU following pegloticase discontinuation although a general increase in SU over time was observed (Fig. 4). eGFR decreased after pegloticase discontinuation but decreases in eGFR were not associated with days since pegloticase discontinuation (not shown).

Because almost all patients (77.3%) had an oral ULT prescribed within the preceding 12 months that presumably could have been refilled following pegloticase discontinuation, changes in SU were not able to be stratified by the subsequent oral ULT therapy nor by its dose. However, 10 patients had no record of receiving an oral ULT prior to pegloticase treatment or following pegloticase discontinuation. Of these 9 patients had laboratory measures with a lower median (IQR) post-pegloticase discontinuation SU compared to the overall cohort (2.3 mg/dL [1.5, 7.5] vs. 5.8 mg/dL [3.5, 8.3]). The restarting of ULTs after pegloticase discontinuation did appear to be restricted by renal function (eGFR < 30 ml/min/1.73 m^2^: 57.1% vs. eGFR ≥ 30 ml/min/1.73 m^2^: 86.1%). Further, median (IQR) eGFR was lower in those without ULT use before or after pegloticase therapy (54.8 [39.2, 72.9] vs. 77.5 [56.4, 96.2] ml/min/1.73 m^2^).

## Discussion

Pegloticase can markedly lower SU, but patients frequently develop ADAs in the absence of concomitant immunomodulatory therapy, limiting urate-lowering efficacy and increasing the risk of IRs [5, 12, 20]. Although patients in this study commonly achieved an SU < 1 mg/dL while on pegloticase, on-treatment increases in SU were common, as was subsequent discontinuation. SU increases were common after pegloticase discontinuation, but we did not see large shifts in eGFR, ESR, or CRP following discontinuation. Concerningly, 9.5% of patients did not start any ULT after pegloticase discontinuation during the observation period.

Our study found that after the second pegloticase infusion, median (IQR) SU was 0.9 mg/dL. However, the upper quartile of 6.6 mg/dL indicated that many patients had a loss of SU-lowering efficacy early in treatment, as also observed in pegloticase clinical trials. For example, in the phase 3 studies of pegloticase monotherapy (i.e., without concomitant immunomodulation), 97% of those who lost response to pegloticase before trial completion, lost SU-lowering efficacy within the first 4 months of treatment [11]. This relatively early loss of SU-lowering response has been associated with ADA development, highlighting rapid development of ADAs in many patients [21]. Of note, only a minority of patients (30.7%) in the current real-world cohort used concomitant immunomodulatory co-therapy, as the time period examined mostly pre-dated the literature supporting the use of concomitant immunomodulation (most-commonly methotrexate) to attenuate ADAs [9, 10]. These observations also suggest that some clinicians continue to administer pegloticase when SU rises above 6 mg/dL, in spite of recommended treatment guidelines [22].

The literature on gout treatment, both XOI and uricosuric, received after discontinuing pegloticase is limited. In our study, and despite our intentionally conservative discontinuation gap of > 12 weeks, 4.5% of patients (*n* = 16) were retreated with pegloticase after initial pegloticase course. Retreated patients achieved a median SU of 0.9 mg/dL (IQR: 0.2, 9.7) after at least 30 days pegloticase re-treatment, with 58.3% meeting the treatment target SU < 6 mg/dL. These findings are in agreement with prior studies. A small open-label extension study of phase 3 pegloticase trial participants evaluated the effect of a pegloticase gap in therapy (> 28 days between infusions) [23]. The mean gap in therapy was 72.5 days, with 10/14 (71.4%) patients achieving an SU < 6 mg/dL. Logistic analysis did not show an effect of gap length (34–167 days) on pegloticase efficacy, but study numbers were small [23]. Additionally, a case series following four patients with a gap in pegloticase treatment found successful SU-lowering in 3/4 (75.0%) patients restarting pegloticase after a gap of 4-147 weeks (initial pegloticase course 22–124 weeks; all patients had SU < 1.5 mg/dL prior to the treatment gap) [24]. In contrast, a small open-label clinical trial in patients with prior loss of SU-lowering response to pegloticase monotherapy showed little re-treatment success with pegloticase plus methotrexate (25 mg subcutaneous) with 1/11 [9%] patients achieving SU < 6 mg/dL during treatment month 6 [25]. Therefore, successful re-treatment occurred in 75% of patients with SU-lowering at initial pegloticase discontinuation, but only 9% of patients with prior loss of response had re-treatment success. This suggests that ADAs against pegloticase are difficult to overcome, even after time and in the presence of immunomodulation. The varying responses to pegloticase retreatment indicate the need to further explore the reasons patients initially discontinue pegloticase and examine retreatment in a larger sample size. Because clinical trial data about re-treatment and gaps in pegloticase therapy are limited, patients re-treated with pegloticase following a gap of > 4 weeks in treatment should be monitored closely due to an increased risk of infusion reactions, including anaphylaxis [6].

The literature regarding long-term renal trends and inflammatory markers from before, during, and after pegloticase therapy is limited. Following pegloticase discontinuation, we observed small decreases in CRP and ESR in post-discontinuation laboratory values compared to on-treatment laboratory values. However, only 5% of patients had sufficient inflammatory biomarker data, likely introducing a bias towards patients needing these measurements for reasons unrelated to gout. We also evaluated eGFR over the study period to examine renal function in patients receiving pegloticase. This is of importance because many gout patients have comorbid CKD, which can limit the use of both oral ULTs and gout flare treatment. The current study showed a minimal post-treatment change in eGFR following pegloticase discontinuation. Previous studies have primarily focused on eGFR during pegloticase treatment and most changes in eGFR measures were not meaningful when comparing pre-treatment to on-treatment values. Prior case series and post hoc clinical trial analyses have shown renal function stability in the majority of patients during 6 months or more of pegloticase treatment [26–28]. This finding remained true both in the presence and absence of methotrexate co-therapy and in patients with and without pre-pegloticase CKD (eGFR < 60 ml/min/1.73 m2) [27, 28].

There are several strengths to this study. First, this study is one of the largest and first to use real-world data to examine patient outcomes following pegloticase discontinuation. Previous studies have been limited by small sample sizes (≤ 14 patients) and only included clinical trial patients. Second, our study population and findings are representative of pegloticase-treated gout patients and how they are managed by community rheumatologists who participate in this large national registry. Finally, the availability of laboratory values allowed us to explore the proportion of patients able to maintain SU < 6 mg/dL following pegloticase discontinuation and subsequent oral ULT initiation. This study also had several limitations. First, a significant portion of the laboratory values were missing, particularly for inflammatory biomarkers. Because all clinical laboratory testing was done as part of standard-of-care, these data may have been skewed towards patients with a higher comorbidity burden. Additionally, not all rheumatology practices contributing RISE data had lab results available within the registry. Second, post-pegloticase SU measurement timing varied and may not have fully captured each patient’s full response to post-pegloticase oral ULTs. Third, medication data were derived from EHR sources based on prescribing information. Given that gout patients have high levels of medication non-compliance, medication prescription may not fully represent medication usage [29]. Thus, assessment of patients’ actual oral ULT use post-pegloticase discontinuation may be misclassified because adherence could not be confirmed. Additionally, 77.3% of patients were prescribed an oral ULT ≤ 1 year before pegloticase initiation and may have refilled the medication following pegloticase discontinuation. This limitation may have led to an underestimation of post-pegloticase oral ULT use and would have made analyses stratified by specific post-pegloticase oral ULT prescription unreliable. Fourth, RISE data is obtained predominantly from community rheumatology practices, and results of the current study may not be generalizable to patients from academic and other subspecialty clinics. Practices may vary among clinic types and directionality of the results would be purely speculative. Fifth, the reasons for pegloticase discontinuation (or interruption) were not known and were assumed to reflect a loss of urate-lowering response. Patients may have also completed their intended course of therapy or simply been lost to follow-up. Finally, due to limitations in available data, we could not capture flare frequency, the number of joints affected by gout, or IRs associated with pegloticase.

## Conclusion

Though optimum gout management following pegloticase treatment remains unknown, we found that most patients (86%) began an oral ULT, with more than half achieving an SU < 6 mg/dL. Patients frequently switched first to allopurinol or febuxostat, but further gout medication changes were common amongst these patients. Additionally, some patients who were able to restart pegloticase after a prolonged gap in therapy achieved its expected SU-lowering effect, although the context for the interruption needs to be further explored. Given that pegloticase is most-often used in patients refractory to oral ULT, this finding is important. This study offers some of the first insight on post-pegloticase gout management, but further study is needed to determine optimal treatment regimens and the possible use of pegloticase re-treatment.

## Data Availability

The data used in this analysis were from the ACR RISE registry and subject to the ACR’s governance policies regarding access. See https://rheumatology.org/request-rise-data for more information.

## Abbreviations

ACR: American College of Rheumatology
ADA: Anti-drug antibody
BMI: Body mass index
CKD: Chronic kidney disease
CRP: C-reactive protein
eGFR: Estimated glomerular filtration rate
EHR: Electronic health record
ESR: Erythrocyte sedimentation rate
FDA: US Food and Drug Administration
GPI: Generic product identifier
HCPCS: Healthcare Common Procedure Coding System
ICD: International classification of disease code
IMM: Immunomodulator
IQR: Interquartile range
IR: Infusion reaction
LOESS: Local regression locally estimated scatterplot smoothing
NDC: National drug code
NLM: National Library of Medicine
NSAID: Nonsteroidal anti-inflammatory drug
RISE: Rheumatology Informatics System for Effectiveness
RXCUI: Prescription concept unique identifier
SD: Standard deviation
SU: Serum urate
ULT: Urate-lowering therapy
XOI: Xanthine oxidase inhibitor
